# Implications of GABAergic Neurotransmission in Alzheimer’s Disease

**DOI:** 10.3389/fnagi.2016.00031

**Published:** 2016-02-23

**Authors:** Yanfang Li, Hao Sun, Zhicai Chen, Huaxi Xu, Guojun Bu, Hui Zheng

**Affiliations:** ^1^Fujian Provincial Key Laboratory of Neurodegenerative Disease and Aging Research, Institute of Neuroscience, College of Medicine, Xiamen UniversityXiamen, China; ^2^Neurodegenerative Disease Research Program, Sanford-Burnham Medical Research InstituteLa Jolla, CA, USA; ^3^Department of Neuroscience, Mayo ClinicJacksonville, FL, USA; ^4^The Interdepartmental Program of Translational Biology and Molecular Medicine, Huffington Center on Aging, Baylor College of MedicineHouston, TX, USA

**Keywords:** GABAergic neurotransmission, amyloid beta-peptides, tau proteins, apolipoproteins E, neuronal inhibition

## Abstract

Alzheimer’s disease (AD) is characterized pathologically by the deposition of β-amyloid peptides (Aβ) and the accumulation of neurofibrillary tangles (NFTs) composed of hyper-phosphorylated tau. Regardless of the pathological hallmarks, synaptic dysfunction is widely accepted as a causal event in AD. Of the two major types of synapses in the central nervous system (CNS): glutamatergic and GABAergic, which provide excitatory and inhibitory outputs respectively, abundant data implicate an impaired glutamatergic system during disease progression. However, emerging evidence supports the notion that disrupted default neuronal network underlies impaired memory, and that alterations of GABAergic circuits, either plays a primary role or as a compensatory response to excitotoxicity, may also contribute to AD by disrupting the overall network function. The goal of this review is to provide an overview of the involvement of Aβ, tau and apolipoprotein E4 (apoE4), the major genetic risk factor in late-onset AD (LOAD), in GABAergic neurotransmission and the potential of modulating the GABAergic function as AD therapy.

## Introduction of Alzheimer’S Disease

Alzheimer’s disease (AD) is the most common age-associated neurodegenerative disorder, which is characterized by the deterioration of memory and cognition. About 10% of the population over the age of 65 and 30–50% of the population over the age of 85 suffer from AD (Querfurth and LaFerla, 2010). Despite significant research and drug development effort in the past decades, currently there are no effective therapies that can prevent, delay or stop the progression of AD, causing a severe burden for the patients, their families and the society.

A small subset (less than 2%) of AD cases result from dominantly inherited genetic mutations in genes encoding the β-amyloid precursor protein (*APP*) and presenilins (*PSEN1* and *PSEN2*, Goate et al., 1991; Levy-Lahad et al., 1995; Sherrington et al., 1995). These AD cases usually develop disease before the age of 60, referring as early-onset familial AD (FAD). Sporadic or late-onset AD (LOAD) usually develops the disease later in life, representing the majority of AD cases (Kanekiyo et al., 2014). The pathological hallmarks of AD include widespread neuronal degeneration, senile plaques and intracellular neurofibrillary tangles (NFTs; Glenner and Wong, 1984; Querfurth and LaFerla, 2010; Tapia-Rojas et al., 2015).

### β-Amyloid

The extracellular senile plaques are composed of accumulated small peptides called β-amyloid (Aβ) derived from the sequential cleavage of APP. There are three major isoforms of APP resulting from alternative splicing, named as APP695, APP751 and APP770 according to their number of amino acid residues. The isoform APP695 is predominantly expressed in neurons and lacks a 56 amino acid Kunitz Protease Inhibitor (KPI) domain at extracellular region (Goate et al., 1991; Zhang et al., 2012; Guzmán et al., 2014; Gautam et al., 2015). Full-length APP is a type I transmembrane protein and can undergo sequential proteolytic cleavage by distinct α-, β- or γ-secretase.

Depending on whether there’s toxic Aβ generation, the APP proteolytic cleavage is divided into two types: amyloidogenic processing and nonamyloidogenic processing. In amyloidogenic processing, APP is first cleaved by β-secretase (beta-site APP cleaving enzyme 1, BACE1), releasing a soluble ectodomain called sAPPβ. The remaining membrane associated carboxyl terminal fragment (βCTF) will be further cleaved by γ-secretase within the cell membrane, releasing neurotoxic Aβ peptides and amyloid intracellular domain (AICD; Li et al., 2014; Ohki et al., 2014; Jung et al., 2015; Neumann et al., 2015; Sadleir et al., 2015; Zhang et al., 2015). The exact cleavage site of γ-secretase may vary inside membrane, yielding Aβ peptides with 36–43 amino acids. Among them, Aβ40 is the major form while Aβ42 is the more amyloidogenic and toxic form (Querfurth and LaFerla, 2010; Zhang et al., 2012; Buggia-Prévot et al., 2014). The AICD tail released inside cytoplasm has been demonstrated to target the nucleus and regulate gene transcription activity (Querfurth and LaFerla, 2010).

As imbalance between production and clearance occurs, Aβ peptides could spontaneously self-aggregate into soluble oligomers, or further grow into insoluble fibers and finally amyloid plaques. The “amyloid hypothesis” is based on the idea that the accumulation of Aβ may be the initiating factor of AD pathogenesis. Multiple lines of evidence have indicated that accumulation of Aβ lead to a neurodegenerative cascade, resulting in synaptic dysfunction, NFT formation and eventually neuronal loss in vulnerable brain regions including cortex and hippocampus (Selkoe, 1998; Hu et al., 2014; Stancu et al., 2014). Compared to insoluble fibers, the soluble Aβ oligomers are more neurotoxic and confer the most deterious effect of Aβ (Querfurth and LaFerla, 2010; Zhang et al., 2012; Tu et al., 2014; Xu et al., 2015).

The nonamyloidogenic processing of APP initiates from the proteolytic cleavage by α-secretase, releasing a soluble sAPPα (Postina, 2012; Jiang et al., 2014; Wang et al., 2014). It is 16 amino acids bigger than sAPPβ because the cleavage site of α-secretase is within the Aβ domain, therefore excluding the possibility of Aβ generation. The membrane remaining αCTF could be further cleaved by γ-secretase, releasing the shorter P83 peptide and AICD. In contrast to Aβ, sAPPα showed important protective roles in neuronal survival and synaptic plasticity against Aβ (Mattson et al., 1993; Goodman and Mattson, 1994; Yamamoto et al., 1994; Furukawa and Mattson, 1998).

### Tau

The major component of AD hallmark NFTs was revealed to be abnormally hyperphosphorylated microtubule-associated protein tau (MAPT), which is essential for assembly and stabilization of microtubules (Spillantini and Goedert, 1998; Querfurth and LaFerla, 2010). The encoding gene of MAPT is located on chromosome 17 in human and expresses six isoforms by alternative splicing in central nervous system (CNS). As a result, the six tau isoforms possess variable N-terminal repeats (0, 1 or 2N) and C-terminal microtubule-binding domains (3 or 4R; Kolarova et al., 2012; Caillet-Boudin et al., 2015; Song et al., 2015).

In physiological conditions, tau is very soluble and mainly located in neuronal axons, where it binds microtubules and regulates the axonal transportation for vesicles and organelles. Since many amino acid residues of the tau protein are potential phosphorylation sites (Ser, Thr, or Tyr), tau is highly phosphorylation-labile (Hasegawa et al., 1992; Hanger et al., 2009). It dynamically switches between phosphorylated and dephosphorylated state during each cell cycle (Pedersen and Sigurdsson, 2015; Wang et al., 2015). It has been demonstrated that increasing tau phosphorylation reduces its affinity for microtubules (Iqbal et al., 2005). The imbalance between tau kinase and phosphatase activities under phathological conditions could lead to tau hyperphosphorylation, which makes tau insoluble and self-aggregate into paired helical filament structure of NFTs. In NFTs, at least 7–8 residues were phosphorylated (Hasegawa et al., 1992; Hanger et al., 2009; Mandelkow and Mandelkow, 2012; Marttinen et al., 2015). Hyperphosporylated tau lacks the affinity for microtubules, therefore making microtubules unstable and impairing their critical function in axonal transportation, eventually resulting in synaptic dysfunction.

The specific tau pathology was reported to correlate well with cognitive abilities. In the cerebrospinal fluid (CSF) of AD patients, the levels of both total tau and phosphorylated tau were found increased (Jack et al., 2013). Besides AD, NFTs composed of hyperphosphorylated tau was found to be a common pathological feature in a number of neurodegenerative disorders including Parkinson’s disease, frontotemporal dementia and progressive supranuclear palsy (PSP), referring as a class of neurodegenerative diseases called tauopathies (Spillantini and Goedert, 1998; Lonskaya et al., 2014; Golovyashkina et al., 2015; Yamada et al., 2015). Considering the critical contribution of tau to the pathological progression of AD, a tau based hypothesis for AD has received wide notice. It emphasizes that the intracellular aggregation of hyperphosphorylated tau leads to the disassembly of microtubules, collapse of synapses, and eventually the cell death in AD (Pedersen and Sigurdsson, 2015).

## Introduction of GABAergic Neurotransmitter System

γ-aminobutyric acid (GABA) is the principle inhibitory neurotransmitter in mammalian CNS. The inhibitory effect of GABA can be conferred through three distinct receptor subfamilies named GABA_A_, GABA_B_ and GABA_C_ receptors. Both GABA_A_ and GABA_C_ receptors are ligand-gated chloride (Cl^−^) channels, whereas GABA_B_ receptors are G-protein coupled metabotropic receptors (Chebib and Johnston, 1999; Bormann, 2000). In the vertebrate brain, GABA_A_ receptors mediate the majority of fast inhibition in the brain. They are composed of five distinct subunits pentamerically assembled, forming a ligand-gated Cl^−^ ion channel. According to their gene identity, the identified GABA_A_ receptor subunits are classified as α1–6, β1–3, γ1–3, ρ1–3, θ, δ, π and ε. In the mammalian brain, the most common combination of GABA_A_ receptor contains two α, two β and one γ subunits. GABA_C_ receptors are composed of ρ1–3 subunits, form homomeric or heteromeric channels, making them distinct from GABA_A_ receptors in pharmacology and function (Johnston, 1994; Lüscher and Keller, 2004). GABA_A_ receptors are widely expressed in all the CNS, while GABA_C_ receptors are highly enriched in retina. Since both GABA_A_ and GABA_C_ receptors are ligand-gated Cl^−^ channels, sometimes we also consider GABA_C_ receptors as a minor subgroup in GABA_A_ receptors (Barnard et al., 1998). GABA_B_ receptor is a metabotropic receptor coupling with G_i/o_ protein. It regulates neuronal activity by either opening the K^+^ channel or inhibiting Ca^2+^ channel via the G_i/o_ protein-dependent signaling cascade (Bowery, 1989; Marshall et al., 1999).

In CNS, the inhibitory action of GABA can be broadly divided into two classes: phasic inhibition and tonic inhibition (Farrant and Nusser, 2005; McQuail et al., 2015). In GABAergic interneurons, neurotransmitter GABA is synthesized from glutamate by the enzyme glutamic acid decarboxylase (GAD). Synthesized GABA is transported along the axon to presynaptic terminals and recruited into vesicles by the vesicular GABA transporter (vGAT; Glykys and Mody, 2007; Gonzalez-Burgos et al., 2009). Upon membrane depolarization induced by action potential, neurotransmitter GABA can be released from presynaptic vesicles into the synaptic cleft, resulting in the burst increase of GABA concentration in the cleft. Most of the released neurotransmitter GABA transiently activates specific GABA receptors on postsynaptic membrane and results in the phasic inhibition of postsynaptic neurons. The phasic inhibition has been demonstrated to be mainly mediated by GABA_A_ receptors contains γ2 subunits postsynaptically (Schweizer et al., 2003; Farrant and Nusser, 2005). Tonic inhibition refers to the sustained form of inhibition upon neuronal cells. Beyond binding to postsynaptic receptors, released neurotransmitter GABA also spills over from synaptic cleft and activates GABA receptors at extrasynaptic area. Tonic inhibition has been demonstrated to be mainly mediated by extrasynaptic GABA_A_ receptors containing π subunit in most brain regions, and GABA_A_ receptors containing α5 subunit especially in hippocampus (Glykys et al., 2008). Recently evidence also indicates the participation of astrocytes and GABA_B_ receptors in tonic inhibition. Especially under pathological conditions, reactive astrocytes release GABA through Bestrophin 1 (Best1) channel. The released GABA could activate both GABA_A_ and GABA_B_ receptors at extrasynaptic area and confer inhibitory effect (Wu et al., 2014).

## GABAergic Neurotransmission in the Pathogenesis of AD

### Alteration of GABAergic Neurotransmission in AD

**Figure 1:**
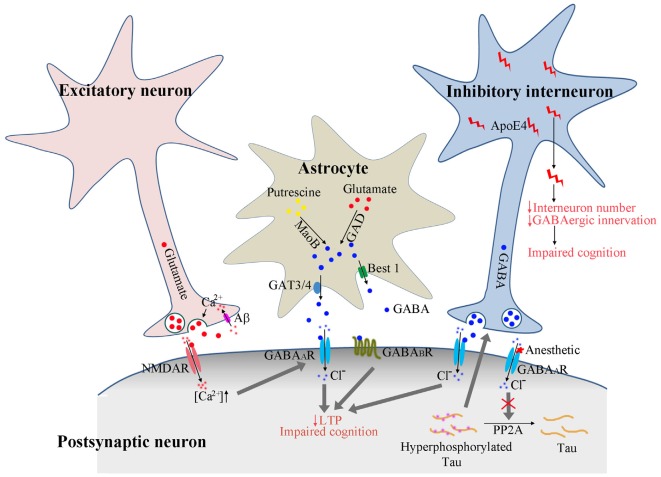
**Proposed model of GABAergic signaling in AD pathogenesis.** Calcium enters the presynaptic terminal via Aβ formed pores on cell membrane. The increased calcium concentration triggers presynaptic glutamate neurotransmitter release and activates postsynaptic receptors. The activated NMDA receptors further enhance the GABA_A_ receptor activation to dampen the overexcitation. In astrocytes, GABA could be synthesized from putresine or glutamate and released via GAT3/4 or Best1 channel. The release of GABA from astrocytes may be enhanced under AD conditions to activate the extrasynaptic GABA_A_ and GABA_B_ receptors, resulting in suppressed long-term potentiation (LTP) and impaired cognition. The activation of GABA_A_ receptors by anesthetics reduces PP2A binding with tau protein, resulting in tau hyperphosphorylation, which feeds back and enhances the activation of GABAergic interneurons and GABA release. ApoE4 secreted from GABAergic interneurons results in reduced interneuron number and reduced GABAergic innervation to other neurons, eventually leading to the disruption of neuronal circuitry and impaired cognition.

In the past, considerable research has focused on the mechanisms of calcium permeable excitatory acetylcholine receptor or glutamate receptors including NMDA and AMPA receptors in AD. Compared to the marked deficits seen in excitatory cholinergic and glutamatergic systems, much less consistent results were revealed for GABAergic system, the main inhibitory neurotransmission in brain. Early studies in postmortem human brains or using animal models concluded that GABAergic neurons and receptors appear more resistant to AD pathology, with only modest loss in AD (Rossor et al., 1982). However, during recent years this statement has been challenged with accumulating evidence, indicating that GABAergic neurotransmission also undergoes profound pathological changes in AD and may be a promising therapeutic target for this neurodegenerative disorder (Figure 1).

By using HPLC, the concentrations of different neurotransmitters were measured in the brain of AD patient samples and age-matched control subjects. In the temporal cortex of AD patients, significantly lower levels of GABA and glutamate neurotransmitters were observed, indicating deficient synaptic function and neuronal transmission in AD (Gueli and Taibi, 2013). The decreased GABA neurotransmitter levels were also observed in the CSF of AD patients and normal humans with aging (Bareggi et al., 1982; Zimmer et al., 1984; Grouselle et al., 1998). An immunocytochemistry study showed diminished perisomatic GABAergic terminals in brain sections from both AD patients and APP/PS1 transgenic mice, especially on cortical neurons adjacent to amyloid plaques, implicating the loss of GABAergic neuronal function in AD (Garcia-Marin et al., 2009; Ramos-Miguel et al., 2015). However, the alteration of synaptic function in AD appears more complicated during the disease progression. By using 4-month old tgCRND8 and 18-month old APP/PS1 transgenic mice respectively, both glutamatergic and GABAergic presynaptic terminals were found elevated at early stage, but declined at late time point in the distinct AD mice models (Bell et al., 2003, 2006; Bell and Claudio Cuello, 2006; Marttinen et al., 2015).

In the nervous system, maintaining a proper dynamic balance between the excitatory glutamate and inhibitory GABA neurotransmitters is critical for neuronal function. Altered synaptic balance was found to be one of the pathological factors that contribute to neuronal disorders including AD, Huntington’s disease and schizophrenia (Kehrer et al., 2008; Cummings et al., 2009; Sun et al., 2009). Aβ the most well-studied neurotoxic factor in AD pathogenesis, has been demonstrated to be a pore-forming molecule. Similar to other pore-forming neurotoxins, Aβ treatment induced perforation in cell membrane, causing a rapid increase of calcium influx in cultured hippocampal neurons (Parodi et al., 2010; Sepulveda et al., 2010). The increased intracellular calcium concentration triggered the presynaptic neurotransmitter release, leading to disrupted neuronal excitation. In the presence of low concentration of Aβ, the frequency of electrophysiological recorded miniature currents increased quickly, but decreased gradually after a couple of hours, indicating the presynaptic vesicular depletion caused by Aβ (Parodi et al., 2010).

A subset of AD patients have been reported to suffer from epilepsy, which is a typical disorder resulting from imbalanced neuronal excitation. In a study with transgenic human APP (hAPP) mice, Aβ was demonstrated to cause aberrant neuronal overexcitation and spontaneous nonconvulsive seizure activity in cortical and hippocampal networks, the most vulnerable brain regions in AD. The increased epileptic activities in turn triggered downstream alterations including GABAergic sprouting and increased synaptic inhibition in hippocampal circuits. These alterations are characterized as compensatory inhibitory mechanisms to ameliorate neuronal overexcitation and keep the normal neuronal excitation (Palop et al., 2007). The finding of direct crosstalk between postsynaptic glutamate NMDA receptors and GABA_A_ receptors further supported the neuronal circuit remodeling mechanisms. In hypothalamic neurosecretory neurons, the NMDA receptor activation by endogenous glutamate was observed to evoke a transient and reversible enhancement of postsynaptic GABA_A_ receptors (Potapenko et al., 2013). The inter-receptor crosstalk between NMDA receptor and GABA_A_ receptor was considered as a compensatory mechanism for dampening the overexcitation commonly observed in pathological conditions. While on the other hand, the increased inhibitory function mediated by GABAergic synapses may interfere with processes required for learning and memory, as indicated by long-term potentiation (LTP) deficits in dentate gyrus (Palop et al., 2007). Consistent with this result, application of GABA_A_ receptor antagonist picrotoxin was demonstrated to prevent such LTP deficits observed in animal model of AD (Kleschevnikov et al., 2004).

Alteration in postsynaptic GABA_A_ receptors was also observed along with AD pathology. Immunohistochemistry study indicates that the β2/3 subunit was markedly preserved, while α1 and γ subunits were upregulated in human AD subjects (Mizukami et al., 1998; Iwakiri et al., 2009). By microtransplating cell membrane isolated from temporal cortices of control and AD patients into Xenopus oocytes, the level of transplanted GABA_A_ receptors were determined by electrophysiological recording. The whole-cell currents mediated by transplanted GABA_A_ receptors were recorded (Limon et al., 2012). A reduction of GABA-evoked currents was observed in cells transplanted with GABA_A_ receptors from AD brains. In particular, the mRNA and protein levels of α1 and γ2 subunits were found down-regulated, whereas α2, β1 and γ1 subunits were up-regulated in AD brains, indicating that GABAergic neurotransmission undergoes a functional remodeling in the cortex of AD patients.

Consistent results for the impact of Aβ on GABA_A_ receptors were obtained in cerebellum. In cultured rat cerebellar granule neurons (CGNs), treatment of recombinant Aβ40, rather than Aβ42, significantly increased the expression level of α6 subunit containing GABA_A_ receptors and their functional recorded currents. In addition, the expression level of α6 protein in APP knockout mice was significantly lower than in WT CGNs. Further investigation demonstrated that Aβ could induce the phosphorylation of ERK and mTOR, resulting in the increased translation of GABA_A_ receptor α6 subunit (Zhan et al., 2014).

The elevated inhibition mediated by GABAergic neurotransmission found in AD mice models do not appear to be consistent with results obtained from AD patients. Even though transgenic mice models have been widely used for AD mechanism investigation, it should be noted that they may not completely represent the complex pathologic characteristics of AD. It should also be noticed that the transgenic mice used in these studies were with various ages, between 4– to 11-month old, at which age even the amyloid plaques were observed, the neuronal death was not detectable (Jo et al., 2014). AD is by far one of the most complicated progressive neurodegenerative disorders. The differential results indicate that at various stages of the disease, GABAergic system might undergo dynamic remodeling and play different roles in AD pathology.

### Alteration of GABAergic Gliotransmission in AD Mice Models

It’s well known that astrocytes are important for uptake and recycling of specific neurotransmitters including GABA and glutamate. In CNS, not only neurons, but also astrocytes were found to be able to produce and release GABA, activating GABA_A_ and GABA_B_ receptors in nearby neurons (Yoon et al., 2012; Yoon and Lee, 2014). Recently, several studies indicate that astrocytes activated by Aβ could release GABA and participate in AD pathology (Mitew et al., 2013; Jo et al., 2014; Wu et al., 2014).

In APP/PS1 and 5× FAD mice, significantly more astrocytes were found activated in hippocampus. Normally astrocytes in wild type mice show minimal GABA immunoreactivity. While in AD mice models, reactive astrocytes were found to abundantly produce and release inhibitory GABA gliotransmitter. As a consequence, HPLC analysis with collected interstitial fluid samples from dentate gyrus revealed significantly elevated GABA level in APP/PS1 mice than wild-type littermates (Jo et al., 2014). In the hippocampus of 5× FAD, the GABA, glutamate and GAD immunostaining intensity were dramatically elevated in astrocytes (Wu et al., 2014). In the synaptosomes isolated from cortex of aged APP/PS1 mice with high amyloid load, the protein level of GAD was found significantly higher than in wild type control and plaque-free region cerebellum. Further study revealed that the increased GAD activity was localized in isolated glial synaptosome, rather than neuronal synaptosome, suggesting that in APP/PS1 transgenic mice, Aβ plaques stimulate the astrocytic GABA synthesis and release (Mitew et al., 2013).

In astrocytes, there may exist more than one pathways for the synthesis and release of GABA. In APP/PS1 mice, the GABA gliotransmitter in astrocytes was demonstrated to be synthesized from putrescine by enzyme monoamine oxidase-B (MaoB) and was released from astrocytes via Best1 channel. The immunoactivity of GABA and MaoB in astrocytes were abnormally and strongly upregulated in the dentate gyrus of APP/PS1 mice and the postmortem brain of AD patients, especially around Aβ plaques (Jo et al., 2014). While in the study with 5× FAD mice, the increased GABA gliotransmitter was synthesized from glutamate by enzyme GAD, and released from astrocytes via the specific GABA transporter GAT3/4 (Wu et al., 2014). The upregulated GABA release from astrocytes could bind to extrasynaptic GABA_A_ and GABA_B_ receptors, strongly inhibit synaptic function and finally leads to the memory and cognitive deficits in AD (Jo et al., 2014).

### Linkage between Tau and GABA_A_ Receptors

GABA_A_ receptor is the most well-known pharmacological target for anesthetics including isoflurane, pentobarbital, propofol and chloral hydrate. In recent years, studies indicate that general anesthesia may contribute to the development and exacerbation of AD (Whittington et al., 2013). Besides Aβ plaques, NFTs composed of hyperphosphorylated tau protein is the most important pathological hallmark of AD. Both pre-clinical and clinical studies have found that anesthesia significantly increase the phosphorylation of tau protein. (Le Freche et al., 2012; Whittington et al., 2013). Since GABA_A_ receptor is the major pharmacological target of most anesthetics, the activation of GABA_A_ receptors was assumed and later confirmed to participate in the anesthesia-induced tau hyperphosphorylation.

By using a live cell reporter system, the direct protein interaction between tau and peptidyl-prolyl cis-transisomerase 1 (Pin1) was identified (Nykänen et al., 2012). Pin1 controls the access of phosphatases to serine-proline or threonine-proline (SP/TP) sites of tau, and therefore promotes dephosphorylation of tau via protein phosphatase 2A (PP2A). Interestingly, with pharmaceutical chemical library screening, several GABA_A_ receptor modulators including anesthetics benzodiazepines and barbiturates were found to increase the interaction between tau and Pin1, but significantly promoted the phosphorylation of tau. Further study revealed that these GABA_A_ receptor modulators do not directly inhibit the activity of PP2A, but recruited more PP2A to cell surface for GABA_A_ receptor β3 subunit dephosphorylation and receptor desensitization, therefore reduced the availability of PP2A for tau dephosphorylation. GABA_A_ receptor activation significantly increased tau phosphorylation at AT8 epitope (Ser199/Ser202/Thr205) in cultured cortical neurons (Nykänen et al., 2012).

On the other hand, the hyperphosphorylated tau also has influence on GABAergic synapses. In tau P301L transgenic mice, in which the extent of tau phosphorylation was remarkably upregulated, the GABAergic interneurons were observed hyperactivated, leading to increased GABA neurotransmitter level in the brain (Nilsen et al., 2013).

Overall, GABA_A_ receptor activation could enhance tau phosphorylation by reducing the association of PP2A with tau, consequently increase the intracellular NFTs in neurons and contribute to the development of AD. *Vice versa*, the hyperphosphorylated tau could enhance GABAergic neurotransmission. There might be a feedback loop between GABA_A_ receptor activation and tau phosphorylation in nervous system (Figure 1).

### Contribution of GABAergic Interneurons in apoE4-Induced Deleterious Effect

Apolipoprotein E4 (apoE4), the major genetic risk factor for AD, accounts for 60–75% of all AD cases, increasing significantly the risk of AD and lowering the age of onset of this disorder (Hu et al., 2015; Liu et al., 2015). By using mice model knockout of endogenous *Apoe* and knockin with various human *APOE* alleles, the neurogenesis was found reduced in both apoE knockout and human apoE4 knockin mice, leading to impaired learning and memory. In apoE4 knockin mice, the GABAergic interneuron number and presynaptic GABAergic input to newly born neurons both decreased, which was associated with increased tau phosphorylation and neurotoxic apoE4 fragments. Treatment with GABA_A_ receptor agonist pentobarbital restored the neurogenesis deficit in apoE4 knockin mice. Consistently, treatment of apoE3 knockin mice with GABA_A_ receptor antagonist picrotoxin decreased the neurogenesis in hippocampus (Li et al., 2009). These findings suggest that the activation of GABA_A_ receptors and GABAergic signaling pathway could be targeted to mitigate the deleterious effects of apoE4 on neurogenesis.

ApoE is expressed in various cell types. In the brain, apoE is mainly released from astrocytes, increasing during aging. It could also be released from neurons, increasing with stress and injury (Huang, 2010; Huang and Mucke, 2012; Mahley and Huang, 2012). Dr. Huang’s group generated specific human *APOE* allele knockin mice models, in which the human *APOE* gene in knockin mice was conditionally deleted in astrocytes, neurons or specific GABAergic interneurons. Deleting apoE4 in neurons, but not in astrocytes, rescued the apoE4-induced deficits including GABAergic interneuron loss and impaired learning and memory. In addition and importantly, conditionally deleting apoE4 in GABAergic interneurons was sufficient to have similar effect and completely prevented the apoE4-induced deficits (Knoferle et al., 2014), suggesting that the apoE4 sourced from GABAergic interneurons is responsible for the deleterious effect of apoE4 on neuronal loss and cognitive deficit.

In human apoE4-positive AD individuals were found often associated with elevated Aβ levels, and Aβ has been shown to impair GABAergic neurotransmission (Huang and Mucke, 2012; Verret et al., 2012). Mice expressing human apoE4 knockin and human APP FAD transgene (apoE4-KI/hAPP-FAD) exhibited high Aβ level and severe cognitive deficit (Palop et al., 2007; Verret et al., 2012). Embryonic interneuron progenitors transplanted into the hilus of apoE4 knockin and apoE4-KI/hAPP-FAD mice successfully developed into mature interneurons that release inhibitory neurotransmitter GABA. In addition, the recovered hippocampal circuitry functionally restored normal learning and memory (Tong et al., 2014), highlighting the importance of GABAergic interneuron and GABAergic neurotransmission in AD pathogenesis.

Interestingly apoE4 also showed sex-dependent characteristics. The risk of developing AD is significantly higher in *APOE4* carrying females than males. Studies found that in female apoE4 knockin mice, the GAD67 or somatostatin positive GABAergic interneuron number decreased in an age-dependent manner, accompanied by spatial learning deficits. The ratio of hilar inhibitory GABAergic interneurons to excitatory mossy cells also decreased. However, in male apoE knockin mice, such ratio was kept consistent, regardless of various apoE genotype and age. Furthermore, in aged male apoE knockin mice, the number of hilar GABAergic interneurons even increased, independent of *APOE* genotype (Leung et al., 2012). These findings suggest that the sex-dependent effect of apoE4 on AD developing risk is at least partially mediated by its differential effects on GABAergic function.

All together, the reported data strongly suggest that GABAergic interneuron plays critical roles in the deleterious effect of apoE4. ApoE4 expressed in GABAergic interneurons may result in apoptosis of these interneurons, leading to reduced GABAergic innervation to other neurons, and disruption of the inhibitory/excitatory balance and neuronal network (Figure 1). The expression of apoE4 was found to cause hyperactivity in human hippocampus (Filippini et al., 2009), which is consistent with the disrupted inhibitory signaling system.

## Potential GABAergic Therapies for AD

Intense therapeutic effort has been taken in the AD field, but the outcomes have been disappointing. As outlined in this review, multiple lines of evidence have strongly suggested that GABAergic neurotransmission plays very important roles in AD pathogenesis. There’s close linkage between GABAergic signaling system and various aspect of AD pathology including Aβ toxicity, tau hyperphosphorylation and apoE4 effect. Accordingly GABA_A_ and GABA_B_ receptor modulators have been investigated in preclinical or clinical tests (Table 1).

**Table 1:** **Effect of GABAergic chemicals in AD models**.

Name	Type	Effect	Reference
Etazolate (EHT-0202)	GABA_A_ receptor agonist	Protected neurons again Aβ-induced toxicity, increased the protein level of sAPPα, displayed anti-inflammation effect after traumatic brain injury and improved cognition in mice models.	Marcade et al. (2008); Drott et al. (2010); Vellas et al. (2011) and Siopi et al. (2008)
Muscimol	GABA_A_ receptor agonist	Inhibited Aβ_25–35_-induced apoptotic death in neurons.	Lee et al. (2005)
Propofol	GABA_A_ receptor agonist	Decreased Aβ generation and accelerated Aβ degradation, reduced the levels of Aβ40 and Aβ42 in aged mice brain. Improved cognitive function and attenuated caspase-3, caspase-9 activation in AD mice model.	Shao et al. (2014) and Zhang et al. (2014)
MRK-016, α5IA, α5IA-II	Inverse agonists of GABA_A_ receptor α5 subunit	Improved cognition in animal models.	Dawson et al. (2006); Atack et al. (2009); Atack (2010) and Guerrini et al. (2013)
CGS9896	Inverse agonists of GABA_A_ receptor α5 subunit	Enhanced the murine memory task.	Guerrini et al. (2009)
Ro-4938581, Ro-4882224	Inverse agonists of GABA_A_ receptor α5 subunit	Reversed the scopolamine-induced impairment in working memory.	Knust et al. (2009)
SGS742 (CGP36742)	GABA_B_ receptor antagonist	Improved attention and working memory in animal models and patients with mild cognitive impairment, increased the levels of NGF and BDNF in rats.	Getova and Bowery (2001); Froestl et al. (2004) and Helm et al. (2005)
CGP55845	GABA_B_ receptor antagonist	Improved cognition in rat model.	Cryan and Kaupmann (2005) and Lasarge et al. (2009)

### GABA_A_ Receptor Agonists

Hyperexcitation of neuronal activity has been observed in AD brain and was considered one of the toxic factors leading to neuronal death. GABAergic neurotransmission was found upregulated in the hippocampus of AD mice models before general cell death (Jo et al., 2014; Wu et al., 2014), which is possibly a neuronal mechanism to neutralize the abnormal hyperexcitation. Some GABA_A_ receptor agonists have been tested and displayed promising effect.

Etazolate, the GABA_A_ receptor modulator, has been shown to exert neuroprotective effect against Aβ toxicity, anti-inflammation after traumatic brain injury and improvement of cognition (Marcade et al., 2008; Drott et al., 2010). Further investigation revealed that etazolate exerted its neuroprotective effect by activating GABA_A_ receptor and stimulating α-secretase cleavage of APP. The neuroprotective effect of etazolate could be fully blocked by GABA_A_ receptor antagonist. Both in rat cortical neurons *in vitro* and in guinea pigs *in vivo*, treatment with etazolate significantly increased the protein level of sAPPα, whose neuroprotective effect has been well demonstrated (Marcade et al., 2008). There might be a relationship between GABA_A_ receptor signaling and the α-secretase cleavage pathway of APP. Etazolate (EHT-0202) has entered Phase II clinical trial for the treatment of AD, with an encouraging result on safety and patient tolerance (Vellas et al., 2011).

Beneficial results have also been obtained with two GABA_A_ receptor agonists, muscimol and propofol (Shao et al., 2014; Zhang et al., 2014). In cultured rat cortical neurons, pretreatment of muscimol significantly inhibited Aβ_25–35_-induced neuronal apoptotic death. GABA_A_ receptor antagonist bicuculine completely blocked the neuroprotective effect of muscimol (Lee et al., 2005). Chronic treatment of aged mice (18-month old) with propofol reduced the levels of Aβ40 and Aβ42 in brain tissue. In addition, decreased expression of BACE1, the critical enzyme for Aβ generation, and increased level of neprilysin, the primary enzyme for Aβ degradation, were both observed after propofol treatment (Zhang et al., 2014), indicating that chronic activation of GABA_A_ receptor by propofol plays neuroprotective role against Aβ by decreasing Aβ generation and accelerating Aβ degradation. Further investigation showed that propofol treatment also improved cognitive function and attenuated caspase-3, -9 activation in both WT and APP/PS1 mice (Shao et al., 2014). All these results support the notion that GABA_A_ receptor activation might have neuroprotective function against Aβ and could effectively improve cognitive function.

### Inverse Agonists of GABA_A_ Receptor α5 Subunit

GABA_A_ receptor is a pentamer containing various allosteric binding sites. It has been suggested that different subunits of GABA_A_ receptor may exert relatively distinct function. For example, α1 subunit is mainly responsible for the sedative action of diazepam, α2 subunit mediates the anxiolytic-like action, whereas α5 subunit may be associated with cognition and memory (Gabriella and Giovanna, 2010). It’s reported that α5 subunit deficiency enhanced hippocampus-dependent memory and spatial learning ability in mice (Collinson et al., 2002; Crestani et al., 2002). In addition, GABA_A_ receptors containing α5 subunit was found upregulated in the dentate gyrus of 5× FAD mice, and has been suggested to mediate the tonic inhibition in CNS. A series of compounds have been developed, serving as inverse agonists of GABA_A_ receptor α5 subunit, which bind to α5 subunit with much higher affinity than other subunits, but negatively regulate receptor activity.

In the early 2000s, the Merck Sharp and Dohme identified a series of benzothiophene derivatives with a notable binding selectivity for GABA_A_ receptor α5 subunit. Among them the ligands MRK-016, α5IA and α5IA-II, all of which displayed encouraging effect on cognition in animal models. The compound α5IA has further advanced to preclinical and clinical studies (Dawson et al., 2006; Atack et al., 2009; Atack, 2010; Guerrini et al., 2013).

Another series of pyrazolo [5, 1-c] [1, 2, 4] benzotriazine 5-oxide (CGS9896) derivatives that are closely correlated to α5IA-II also showed important activity in enhancing the murine memory task (Guerrini et al., 2009). Other two compounds Ro-4938581 and Ro-4882224 from Hoffmann-La Roche Company were shown to significantly reverse the scopolamine-induced working memory impairment. Supported by this result, these two compounds Ro-4938581 and Ro-4882224 have been selected as candidates for further clinical studies (Knust et al., 2009).

### GABA_B_ Receptor Antagonists

In AD mice models and human AD patients, GABA released from activated astrocytes was significantly increased. The released GABA could bind to neuronal GABA_B_ receptors at extrasynaptic area, and in turn participate in the inhibition of synaptic release in APP/PS1 mice (Jo et al., 2014). To alleviate the inhibition of synaptic function and improve the cognition deficit in AD, several compounds served as GABA_B_ receptor antagonists have been tested.

SGS742 (CGP36742) is the first GABA_B_ receptor antagonist tested in clinical trials for AD treatment. In rodents and Rhesus monkeys, SGS742 displayed pronounced cognition enhancing effects in various cognitive and learning tasks. It blocked the inhibitory postsynaptic potential (IPSP) and paired-pulse inhibition (PPI) in hippocampus both *in vitro* and *in vivo*. It also increased the mRNA and protein levels of NGF (nerve growth factor) and BDNF (brain derived neurotrophic factor) in cortex and hippocampus of rats (Froestl et al., 2004). In addition, SGS742 was well tolerated in both experimental animals and human volunteers. In a Phase II study, oral administration of SGS742 for 8 weeks significantly improved attention and working memory in patients with mild cognitive impairment (Getova and Bowery, 2001; Froestl et al., 2004; Helm et al., 2005). These encouraging findings make SGS742 a promising candidate for dementia treatment and pushed it for further clinical test.

CGP55845 is another GABA_B_ receptor antagonist undergoing preclinical study. In an aged rat model with impaired cognition, treatment with CGP55845 completely reversed its olfactory discrimination learning deficits and restored its performance (Cryan and Kaupmann, 2005; Lasarge et al., 2009). These results supported the potential importance of GABA_B_ receptor as the pharmaceutical target in cognition enhancing activities.

## Outlook and Conclusions

Although tremendous understanding of AD pathogenesis has been achieved since last decade, there is still no effective therapy to prevent, delay or stop the disease progression. For a long time, inhibitory GABAergic interneurons and GABA receptors were considered generally preserved in AD, compared to the more vulnerable excitatory glutamate and acetylcholine neurotransmission systems. However, in recent years, abundant evidence has emerged to support the notion that GABAergic signaling system undergoes pathological alterations and contribute to AD pathogenesis. Accordingly, targeting GABAergic neurotransmission is being explored as a potential therapy for AD treatment. However, inconsistent and controversial results have been reported, and these are likely attributed by the complex pathological processes, limitations of the animal models, and differences in the timing and duration of the experimental design. Overall, we hope that this review provides an overall of the current understanding of the role of GABAergic inhibitory neurons in AD and calls for the need of further investigating the GABAergic system in AD pathogenesis using more sophisticated models, rigorous methods and advanced technology. In this review, we present multiple lines of evidence that there is significant GABAergic derangement in AD and that Aβ, tau and apoE4 all mediate GABAergic dysfunction.

## Author Contributions

YL wrote the manuscript. HZ reviewed and edited the manuscript. ZC contributed to the references organization. All authors have read and approved the final manuscript.

## Conflict of Interest Statement

The authors declare that the research was conducted in the absence of any commercial or financial relationships that could be construed as a potential conflict of interest.
